# Surgery for Prosthetic Aortic Valve Endocarditis: How We Teach It

**DOI:** 10.1016/j.atssr.2024.10.006

**Published:** 2024-10-23

**Authors:** Ali Hage, Shinya Unai, Haytham Elgharably, Gösta B. Pettersson

**Affiliations:** 1 Endocarditis Center, Cleveland Clinic, Cleveland, Ohio

Aortic prosthetic valve endocarditis (APVE) is often manifested with complex pathologic changes for which redo operation can be technically demanding. During the last 3 decades, as a tertiary center with high volume of infective endocarditis cases, we developed a standardized approach to teach our trainees a systematic way to manage APVE following the principles described in the current American Association for Thoracic Surgery guidelines for surgical treatment of infective endocarditis.^1^

## Preparation

There are specific basic principles that we teach our trainees when it comes to surgery for APVE.

• “Prepare for the battle!” Expect worse findings and invasive pathologic changes than on preoperative imaging. Be sure you have the skills and tools to do the operation!
• “See what you are doing, know what you are doing!” This refers to how to set up the operation, exposure, and understanding anatomy and pathology.
• “Débride and don’t worry yet about implanting the new prosthesis!” This refers to removing all prosthetic materials and all ill-appearing infected and necrotic tissue.
• “Living on the edge!” This refers to the difficult dissection around the aortic root, especially after previous root replacement, including mobilizing the coronary buttons.
• “Now let’s see what we have!” This refers to the reconstruction required after radical débridement of an invasive pathologic process.
• “We are not done yet!” This refers to the challenging postoperative management of patients after surgery for APVE.

## How We Teach It

### Adequate Preoperative Investigations: “Prepare for the battle!”

Patients with infective endocarditis are managed by a multispecialty team; the core members are infection disease specialists and cardiologists. The cardiac surgeon should be involved early and neurologists and other specialists as needed.

**Figure 1:**
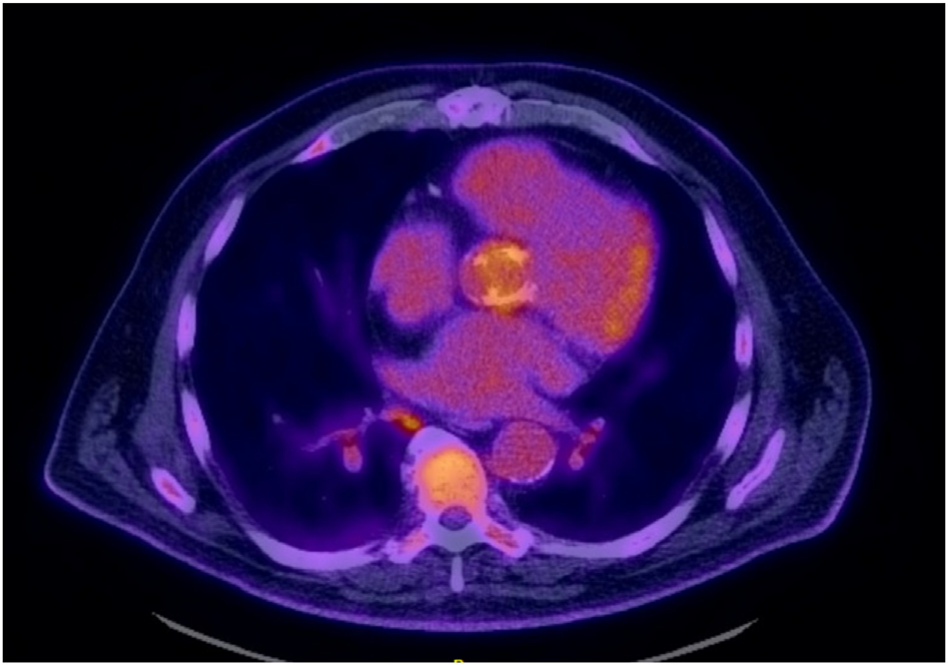
Positron emission tomography scan showing diffuse uptake around the aortic prosthesis suggestive of infection.

Transthoracic echocardiography is the initial study for suspected APVE and can reveal vegetations on the aortic prosthesis, thickening of the leaflets, or evidence of valvular dysfunction. It is commonly complemented by transesophageal echocardiography to better delineate the pathologic process. We teach our trainees how to carefully look for signs of invasive disease, such as periaortic root thickening, abscess cavities, or fistulas between the aortic root and adjacent chambers. A gated computed tomography (CT) angiography scan of the chest, abdomen, and pelvis is performed for several reasons: to better characterize the aortic root disease; to identify distant embolization in the abdomen; to screen for primary and secondary foci of infection; and to prepare for sternal reentry. If a patient presents with renal dysfunction, CT scan is obtained without contrast enhancement. When the findings on echocardiography or CT scan are inconclusive, a positron emission tomography scan is sometimes useful to detect metabolic activity suggesting infection around the aortic prosthetic valve (Figure 1).

For neurologic clearance, asymptomatic patients undergo a baseline CT study without contrast enhancement of their brain to rule out silent cerebrovascular events related to APVE. Patients presenting with neurologic symptoms or complications require evaluation by a neurologist as they may need additional dedicated imaging, which could necessitate intervention or delay of the surgery. If the gated CT angiogram is inconclusive, patients with risk factors for coronary disease need coronary angiography before surgery; large vegetations add risk of embolism and are a relative contraindication. Patients presenting with vascular embolic complications, such as limb ischemia or mesenteric mycotic aneurysm, require evaluation by a vascular surgeon and intervention before cardiac surgery. Patients presenting with splenic abscess require evaluation by a general surgeon; if the process is stable on imaging without signs of impending rupture or bleeding, the cardiac operation has priority and splenectomy can be performed later. The threshold to perform head to toe CT screening to identify extracardiac primary and satellite infections is low. Source control is achieved before cardiac surgery, such as explantation of an indwelling catheter or infected dialysis grafts. Patients with pacemakers or new conduction abnormality require evaluation by an electrophysiologist for plans of surgical extraction during surgery and possible epicardial system placement.

### Set up for Redo Surgery for APVE: “See what you are doing, know what you are doing!”

The trainees are taught to consider the following when planning for redo sternotomy.

1. Is reentry safe? Distance of mediastinal structures to the sternum? Arterial structures in immediate contact with sternum?
  a. If it is not safe: Plan for peripheral cannulation? Need hypothermic circulatory arrest? Is cooling safe? Severity of aortic insufficiency? Fistulas? Alternative strategy?
2. Is it safe for central cannulation? Check for aortic calcifications. Is there enough room for clamping?
3. Is there a previous distal ascending aorta graft that needs to be explanted? Do you need hypothermic circulatory arrest for distal aortic anastomosis? What is the brain protection strategy?
4. What is the myocardial protection strategy? Previous patent bypass grafts?
5. Need for coronary bypass? Conduits?

**Figure 2:**
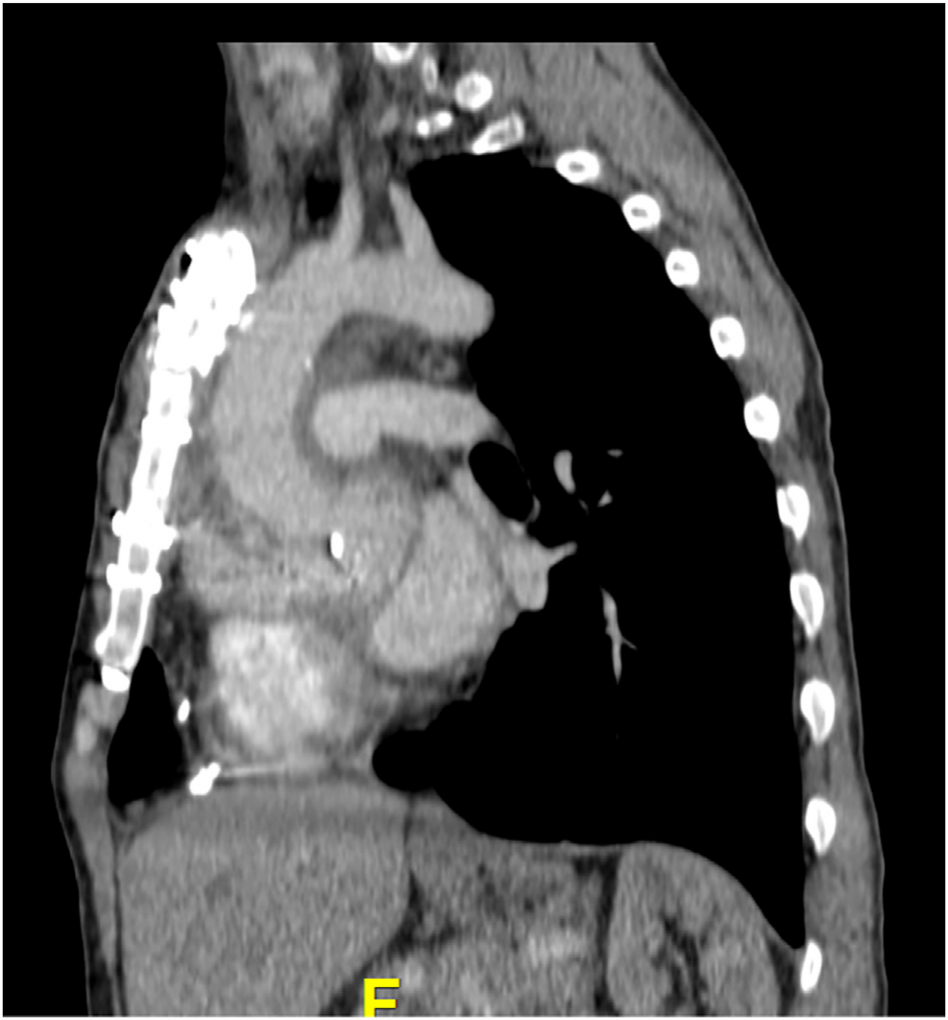
Preoperative computed tomography scan showing proximity of the ascending aorta to the sternum.

The CT scan of the chest is reviewed with the trainees, and we carefully look at the hostility of the chest by assessing the proximity of the sternum to vital retrosternal structures (aorta, innominate vein, right ventricle, previous bypass grafts). If the reentry is not safe (Figure 2), we teach our trainees to obtain peripheral access first (for arterial access, either axillary or femoral artery; avoid femoral in elderly patients). The size and quality of the axillary or femoral artery and of the entire arterial tree are assessed on the CT angiogram of the chest, abdomen, and pelvis. Cardiopulmonary bypass is not routinely initiated before redo sternotomy to minimize time on pump and to allow mediastinal dissection off heparin. Retrosternal aortic pseudoaneurysm adherent to the sternum will require redo sternotomy on cardiopulmonary bypass—the strategy must ensure brain and heart protection as aortic cross-clamping will not be instant! For central cannulation, our preference is aortic and bicaval cannulation as a setup for a possible prolonged case. This will allow placement of a direct retrograde cardioplegia catheter for adequate myocardial protection and access to the right atrium and both tricuspid and mitral valves in cases of invasive disease.

### Explanting the Infected Aortic Prosthesis and Débriding: “Débride and don’t worry yet about implanting the new prosthesis!”

After aortic cross-clamping, cardioplegia, and cardiac arrest, dissection is carried out around the ascending aorta to separate it from the pulmonary artery, superior vena cava, and right atrium. The aortotomy is made at the level of (or just above) the previous aortotomy. The aortic valve prosthesis is inspected. Any loose vegetations are removed and sent for microbiologic and pathologic analysis. The previous valve sutures are exposed, a spatula or a Rongeur forceps is used to remove the pannus, the knots are grabbed, and the sutures are cut and pulled. We then instruct our trainees to use a spatula dissector to carefully push all scar off the aortic prosthesis cuff toward the annulus, thus avoiding creation of defects in the native annulus. After all the valve sutures are removed sequentially, the prosthesis cuff is carefully separated from the annulus circumferentially, avoiding separating intima-media from the adventitia. The annulus is then débrided of all foreign materials and subvalvular pannus. The aortic annulus and root are examined to assess tissue integrity. Abscess cavities need to be aggressively débrided. Any suspicious lesions need to be explored to avoid missing any residual cavities. Fistulous communications are identified and débrided as well. Copious irrigation with saline is performed, the annulus is swabbed with chlorhexidine or other antiseptic solutions, and surgical instruments are changed.

If we are dealing with a previous transcatheter aortic valve replacement, the location of the aortotomy depends on the length of the stent frame. For transcatheter aortic valve replacement valves with long frames, the incision cannot be made above the upper margin of the frame as it will be too far away from the aortic annulus. Therefore, the trainees are taught to palpate the aorta to identify the upper margin of the stent frame and to open the aorta 1 to 1.5 cm below the upper margin of the stent frame. Once the aorta is opened, a spatula dissector is used to carefully create a dissection plane between the stent frame and the aorta, being careful to stay on the stent frame and not into the aorta. Once the stent frame begins to be free, a Kocher forceps is used to grab and squeeze the stent, allowing us to continue our dissection until the stent is freed up circumferentially and can be removed without further damage to the aortic wall. The native aortic valve is excised, and annulus, root, and aorta integrity is examined to decide whether they are healthy enough to allow prosthetic valve replacement or a root replacement is required.

When an aortic root replacement is not required, we do not believe that the choice between a biologic and a mechanical prosthesis has important impact on the recurrence of infection. Biologic valves are preferred in patients presenting with cerebral disease who are at increased risk for perioperative complications to avoid the need for anticoagulation for mechanical valve after surgery. Our preferred prosthesis for invasive disease is an aortic allograft. After previous aortic root replacement, explanting the previous prosthesis and root can be more technically challenging, especially for infected composite grafts owing to the dense scarring including surrounding structures. For previous composite grafts, after dissecting out the root and valve sutures, the graft is explanted, leaving no graft material behind to avoid recurrence of endocarditis. After previous valve-sparing root reimplantation or porcine (Freestyle [Medtronic]) or allograft root replacement, dissection is carried out down to the proximal suture line in the annulus or left ventricular outflow tract (LVOT) to ensure removal of all prosthetic and xenograft and allograft material. Aggressive débridement follows the same principles as described after explantation of an aortic valve prosthesis.

We prefer to open and inspect the right atrium in patients with invasive aortic root endocarditis. In patients with conduction abnormality or heart block, the infection extends into the right atrium and triangle of Koch, ultimately creating a fistula between the aortic root and the right atrium or right ventricle.

### Dissecting out the Aortic Root: “Living on the edge!”

Redo aortic root replacement in the setting of APVE can be technically demanding, especially after previous root replacement, use of hemostatic agents (eg, glue) or pericardial or felt strips, and presence of extensive invasive disease with abscess cavities. The most challenging aspect is usually mobilizing the left coronary button. To overcome this, we instruct our trainees to define the right pulmonary artery and develop the plane between the right pulmonary artery and the left coronary artery. This plane is easier to follow and allows safe dissection and generous mobilization. Still, avoid denuding the pulmonary artery, and if you do, recognize and repair or support defects with pericardial patch. Use a coronary probe to help define the course of the left coronary artery. On occasion, 1 or both coronary buttons are extensively stuck, and use of a Cabrol interposition graft (with either saphenous vein or synthetic graft) between the coronary ostium and the aortic root may be justified.

### Dealing With Surrounding Invasion: “Now let’s see what we have!”

After débridement of all infected and necrotic tissue and explantation of all prosthetic material, it is time to replace the valve and to reconstruct the aortic root. APVE is commonly associated with invasive disease that results in disintegration of the surrounding tissues. Proximal destruction of muscle is unusual; note that even after extensive invasive disease, the LVOT remains intact, maybe apart from the aortomitral continuity and trigones. Our preference is to use an aortic allograft as we believe that the allograft has excellent resistance to reinfection, has the perfect quality for suturing in inflamed tissue, provides the extra tissue including trigones and mitral leaflet needed for reconstruction after aggressive débridement and involvement of the aortomitral continuity, which provides good hemostasis. To perform aortic allograft root replacement, we teach our trainees several important principles. To size the homograft, we measure the LVOT with a Hegar dilator, which provides the true dimension after débridement, and we choose 1 to 2 mm smaller. Preoperative sizing by CT or transesophageal echocardiography is unreliable. Trimming the allograft, we leave 4 to 5 mm of muscle and as much mitral valve leaflet as needed. The homograft is implanted with 3 or 4 running 3-0 or 4-0 monofilament sutures. Bites of the running sutures in the muscular portion of the allograft are taken inside-out the allograft, keeping 1- to 2-mm distance from the aortic allograft valve. The allograft should sit deep in the LVOT, and there should be no longitudinal tension on it. The coronary buttons are reimplanted in the usual fashion, being gentle to the allograft as the sinus tissue is thinner and fragile; if needed, a strip of pericardium for reinforcement is used.

When the infection extends into the intervalvular fibrosa (IVF), extensive débridement of the aortomitral curtain is necessary to control the infection and to prevent recurrence. If the pathologic process is limited to the IVF and base of the anterior mitral leaflet, reconstruction can be achieved with anterior mitral valve leaflet of the aortic allograft; we refer to this reconstruction as a hemi-commando procedure. The mitral valve may require an annuloplasty band. If the mitral valve is destroyed by infection and needs replacement, we perform the commando operation, which includes replacement of both valves and the IVF.

Again, inspect the atrial and ventricular septum from both sides, below the septal leaflet of the tricuspid valve. An identified defect is closed with a pericardium patch, trying to avoid the conduction bundle if the patient does not already have heart block.

Identification of the etiologic organism and its sensitivity is important to the final cure of the infection. Our protocol is to divide the specimens between pathologic and microbiologic examination, and we have a low threshold to ask for molecular testing or polymerase chain reaction analysis to identify the organisms.

### Postoperative Management: “We are not done yet!”

At the end of the procedure, we warn our trainees to not let their guards down as several challenges usually arise and require their utmost attention. These patients are often very sick with coagulopathic after prolonged surgery and require blood product transfusion. For severe coagulopathy, we use a delayed chest closure technique with negative pressure wound therapy. These patients are vasoplegic after prolonged cardiopulmonary bypass and systemic inflammatory activation and often require multiple vasopressor agents. In the setting of significant pulmonary hypertension or right ventricular dysfunction, we tend to use inhaled epoprostenol. Neurologic monitoring is essential postoperatively for all patients, particularly for those who had preoperative strokes or bleeding. The threshold for neurologic consultation and repeated imaging is low. These patients routinely continue intravenous antimicrobials for 6 weeks based on the results of the explanted valve cultures and clinical response.

## Comment

Surgery for APVE can be challenging, especially with extensive invasion and destruction of aortic root tissues.^2^ Microbes establish biofilm structures on prosthetic valves and become more resistant to antibiotics, which necessitates radical débridement and removal of all foreign material.^3^ There is a learning curve for these complex surgeries that requires following basic surgical principles and systematic approach and guidance of an experienced surgeon (Video).
